# Sex-dependent associations of childhood maltreatment with obesity-related traits: results from the German National Cohort (NAKO)

**DOI:** 10.1038/s41366-025-01914-2

**Published:** 2026-02-06

**Authors:** Philipp Töpfer, Johanna Klinger-König, Ulrike Siewert-Markus, Sabine Schipf, Beate Fischer, Anja M. Sedlmeier, Antje Hebestreit, Wolfgang Ahrens, Klaus Berger, Hermann Brenner, Stefanie Do, Jana-Kristin Heise, Stefanie Jaskulski, André Karch, Thomas Keil, Carolina Klett-Tammen, Michael F. Leitzmann, Annette Peters, Börge Schmidt, Matthias B. Schulze, Stefan N. Willich, Marcus Dörr, Henry Völzke, Marcello R. P. Markus, Sylvia Stracke, Hans J. Grabe, Till Ittermann

**Affiliations:** 1 Department of Internal Medicine A, University Medicine Greifswald, Greifswald, Germany; 2 Department of Psychiatry and Psychotherapy, University Medicine Greifswald, Greifswald, Germany; 3 Institute for Community Medicine, Department SHIP Clinical-Epidemiological Research, University Medicine Greifswald, Greifswald, Germany; 4 Department of Epidemiology and Preventive Medicine, University of Regensburg, Regensburg, Germany; 5 Center for Translational Oncology, University Hospital Regensburg, Regensburg, Germany; 6 Bavarian Cancer Research Center (BZKF), Regensburg, Germany; 7 Leibniz Institute for Prevention Research and Epidemiology – BIPS, Bremen, Germany; 8 Institute of Epidemiology and Social Medicine, University of Münster, Münster, Germany; 9 Division of Clinical Epidemiology and Aging Research, German Cancer Research Center (DKFZ), Heidelberg, Germany; 10 Division of Preventive Oncology, German Cancer Research Center (DKFZ) and National Center for Tumour Diseases (NCT), Heidelberg, Germany; 11 German Cancer Consortium (DKTK), German Cancer Research Center (DKFZ), Heidelberg, Germany; 12 Department of Epidemiology, Helmholtz Center for Infection Research (HZI), Braunschweig, Germany; 13 Institute for Prevention and Cancer Epidemiology, Faculty of Medicine and Medical Center, University of Freiburg, Freiburg, Germany; 14 Institute of Social Medicine, Epidemiology and Health Economics, Charité—University Medicine Berlin, Berlin, Germany; 15 Institute for Clinical Epidemiology and Biometry, University of Würzburg, Würzburg, Germany; 16 State Institute of Health I, Bavarian State Office for Health and Food Safety, Erlangen, Germany; 17 Institute of Epidemiology, Helmholtz Zentrum München—German Research Center for Environmental Health (GmbH), Neuherberg, Germany; 18 Institute for Medical Information Processing, Biometry and Epidemiology, Medical Faculty, Ludwig-Maximilians-Universität München, Munich, Germany; 19 German Center for Diabetes Research (DZD e.V.), Neuherberg, Germany; 20 German Center for Mental Health (DZPG), Partner Site Munich, Munich, Germany; 21 Institute for Medical Informatics, Biometry and Epidemiology, University Hospital Essen, University Duisburg-Essen, Essen, Germany; 22 Department of Molecular Epidemiology, German Institute of Human Nutrition, Potsdam-Rehbruecke, Nuthetal, Germany; 23 Institute of Nutritional Science, University of Potsdam, Nuthetal, Germany; 24 Department of Internal Medicine B, University Medicine Greifswald, Greifswald, Germany; 25 German Centre for Cardiovascular Research (DZHK), Partner Site Greifswald, Greifswald, Germany; 26 German Center for Neurodegenerative Diseases (DZNE), Site Rostock/Greifswald, Greifswald, Germany

**Keywords:** Epidemiology, Risk factors

## Abstract

**Background:**

The relationship between childhood maltreatment (CM) and obesity is nuanced, and recent evidence suggests stronger associations between CM and obesity-related traits in females compared to males. This study aims to validate and extend these findings in a large sample from the German National Cohort (NAKO).

**Methods:**

The NAKO is a population-based cohort study including 204,744 adults. For the present analyses, 151,143 individuals (74,596 female) were included. CM was assessed using the Childhood Trauma Screener (CTS). From the CTS, an overall severity score (CTS sum score), a cumulative CM score (number of CM subtypes with at least moderate severity), and five CTS subtypes were considered as exposures. Obesity-related traits included anthropometric (height, weight, body mass index [BMI], waist circumference [WC]) and body fat markers (relative fat mass [rFM], subcutaneous [SAT], visceral adipose tissue [VAT]). Sex-stratified linear and logistic regression models were adjusted for age, education, and examination center to associate CTS-based scores with obesity-related traits.

**Results:**

Associations of the CTS sum score with weight, BMI, WC, rFM, and SAT were stronger in females compared to males, while similar associations were observed for VAT. In both sexes, most obesity-related traits exhibited dose-response relationships with increasing numbers of CM subtypes. Compared to unexposed females, females with exposure to ≥3 CM subtypes had a higher risk for obesity (i.e., BMI ≥ 30 kg/m^2^; OR = 1.56; 95% CI: 1.43, 1.71) and high WC (i.e., WC ≥ 88 cm; OR = 1.39; 95% CI: 1.29, 1.50). In males, exposure to ≥3 CM subtypes was also associated with increased obesity risk (OR = 1.51; 95% CI: 1.32, 1.72) and high WC (i.e., WC ≥ 102 cm; OR = 1.31; 95% CI: 1.18, 1.44). Physical and emotional abuse exhibited the strongest average associations and were associated with the most outcomes.

**Conclusion:**

Associations of CM exposure with adult anthropometric and body fat markers are stronger in females compared to males.

## Introduction

Severe stressors occurring during early developmental periods, such as child abuse and neglect, represent a cluster of risk factors that substantially contribute to morbidity and mortality at the population level [1]. The largest proportion of deaths associated with childhood maltreatment (CM) in the United States are due to heart diseases [1]. Against this background, there is growing evidence that consistently demonstrates that people who were exposed to CM have a higher risk for cardiometabolic diseases such as ischemic heart disease [2], coronary heart disease [3], stroke [4, 5], type-2-diabetes mellitus, and overweight or obesity [4, 6, 7]. Overweight and obesity, which are themselves growing public health concerns [8], are associated with increased morbidity and all-cause mortality risk, mainly due to cardiovascular disease-related deaths [9]. It seems, therefore, plausible that a significant proportion of CM exposure-associated morbidity and mortality can be attributed to the increased obesity-related cardiometabolic risk in these individuals.

Importantly, CM exposure was associated with all-cause mortality in females but not in males in a large US sample [10]. This study highlights the need to adopt a sex and gender-sensitive perspective to investigate long-term health outcomes observed in CM survivors. Indeed, there is now an emerging trend that recognizes the role of sex and gender as major modifiers of health and disease [11]. For example, there is evidence that the association of CM with overweight and obesity is stronger and more consistent in females compared to males [7, 12, 13] and meta-analytical evidence suggests that the association between CM and obesity was stronger in studies that included a higher proportion of females [7]. More recently, we have shown in 4006 adults from the general population that CM exposure was consistently and more strongly associated with a multitude of different obesity-related traits in females than in males [12]. In females, associations of CM exposure and obesity-related traits were mainly driven by exposure to emotional and physical abuse. In males, we observed an association between CM exposure and reduced body height, as well as increased waist-to-height ratio and visceral adipose tissue (VAT). Like in females, these associations were mostly confined to exposure to either childhood emotional or physical abuse.

The purpose of the present study was to validate and extend these findings in a substantially larger sample. Following recommendations for the analyses of sexually dimorphic traits [14], such as anthropometric indicators of overweight/obesity and body fat markers [15, 16], we analyzed the associations of CM exposure with several standard anthropometric indicators, direct measurements of body fat by ultrasound, and bioelectrical impedance measures of relative fat mass (rFM) stratified by sex in the German National Cohort (NAKO).

## Material and methods

### Study population

The NAKO baseline survey was conducted between 2014 and 2019. Age and sex-stratified samples were randomly drawn from compulsory registries of residents within a total of 16 study areas (and 18 study centers) throughout Germany. At baseline, more than 204,744 adults, aged 20–69 years, of the general German population from rural and urban areas were recruited [17]. Further detailed information on sampling, inclusion criteria, and study protocols can be found in Peters et al. [17]. The Ethics Committees of all study sites approved the study. All participants gave written informed consent for participation in the study and data utilization. The NAKO was conducted in accordance with the Declaration of Helsinki. NAKO data and bio samples can be obtained via an electronic application portal (https://transfer.nako.de). Sequential exclusion of participants with missing data (Fig. S1) resulted in a study population of 151,143 individuals.

### Childhood maltreatment

Information about CM exposure was obtained as part of a touchscreen section using the CTS, a brief screening version derived from the Childhood Trauma Questionnaire (CTQ) [18]. The CTS covers five types of maltreatment, one item representing each CM subtype: emotional, physical, and sexual abuse as well as emotional and physical neglect. Frequency ratings for each CTS item are given on a five-point scale (range: 1 = “not at all” to 5 = “very often”). All items were summed up for the CTS sum score, resulting in values ranging between 5 and 25, with higher values indicating more severe CM exposure. In addition, and based on established cut-off values [19], a dichotomous variable was computed to indicate none/mild or moderate/severe CM exposure for each item. Subsequently, an index of cumulative CM exposure was computed from these dichotomous values to capture how many different moderate/severe CM subtypes each individual experienced (i.e., none, one, two, three or more). In addition, a global value captured whether at least one moderate/severe CM was reported, regardless of the subtype.

### Obesity-related traits

Body weight was measured to the nearest 0.1 kg in underwear and without shoes using a digital scale integrated in the medical Body Composition Analyzer (mBCA) 515 (seca GmbH & Co. KG; Hamburg; Germany) [20]. Body height (in cm) was measured with the Stadiometer 274 (seca GmbH & Co. KG; Hamburg; Germany). The BMI was calculated as body weight in kg divided by body height in meters squared. Based on the BMI, we created categorical variables indicating either overweight (25 kg/m^2^ ≥ BMI < 30 kg/m^2^) or obesity (BMI ≥ 30 kg/m^2^). Waist circumference (in cm) was measured with a flexible, non-stretchable graduated tape (Umfangmessband 201, seca GmbH & Co. KG; Hamburg; Germany). To determine waist circumference, the examiner palpated the iliac crest and the lowest rib at the lateral part of the body. Measurements were taken in the middle of these two landmarks. We additionally computed a dichotomous variable based on sex-specific cut-offs for waist circumference (females: ≥88 cm; males: ≥102 cm) as an indicator of central/visceral obesity. WHtR was calculated as waist circumference divided by height. Relative fat mass in % was measured by bioelectrical impedance analysis using the phase-sensitive multifrequency device mBCA 515 (seca GmbH & Co. KG; Hamburg; Germany). SAT and VAT thickness (in mm) were measured by abdominal ultrasound using a Philips iE33 device (Philips GmbH; Hamburg; Germany) with a 5 MHz convex transducer. Further detailed information on data acquisition related to anthropometrics and body fat has been published elsewhere [20].

### Covariables

Information about years of formal education based on the international standard classification of education (ISCED 97) and age were obtained in computer-assisted personal interviews. A categorical variable was created for education, indicating low ( < 10 years), medium (10 years), or high (>10 years) level of formal education. In supplementary analyses, we additionally considered migration background as a covariate. The assignment of migration background (yes/no) was dependent on the nationality and country of birth of participants and their parents, and described in more detail elsewhere [21].

### Statistical methods

Descriptive data are presented as mean and standard deviation (continuous data) or as absolute numbers and percentages (categorical data) stratified by sex and absence (CM−) /occurrence (CM+) of at least one CM of moderate/severe severity. The CTS sum score, cumulative CM exposure, and CTS subscales were associated with obesity-related traits by sex-stratified linear regression models adjusted for age, years of formal education, and examination center. The results of the regression models are reported as *β* coefficients and 95% confidence intervals (CI). The Bonferroni method was applied to correct for multiple testing and *p* values <0.003 (regression models for eight outcomes in two sexes: *p*_corr_ = 0.05/16 = 0.003) were considered statistically significant. Additional explorative analyses also tested age-dependent associations between CTS sum scores and obesity-related traits (only continuous outcomes). For this purpose, fully adjusted sex-stratified regression models included a CTS sum score x age interaction term. We conducted all analyses with STATA 18.0 (Stata Corporation, TX, USA).

## Results

### Sample characteristics

At least one moderate/severe CM was reported by 17,900 males (23.4%) and 21,339 females (28.6%). Of those, 12,565 males and 13,655 females reported one type of CM, 3500 males and 4353 females reported two types of CM, while 1835 males and 3331 females reported three or more types of CM (Table 1). Among males, who reported at least one type of CM, the most frequent CM was physical neglect (42.3%), followed by physical abuse (35.7%) and emotional neglect (28.0%). In females who reported at least one CM, the most frequent CM was physical neglect (34.7%), followed by emotional abuse (34.5%) and sexual abuse (33.6%).

**Table 1:** Characteristics of the study population stratified by sex and childhood maltreatment (CM).

	Males		Females	
	CM−	CM+	CM−	CM+
*N*	58,694 (38.8%)	17,900 (11.8%)	53,210 (35.2%)	21,339 (14.1%)
Age in years	48.5 (12.5)	52.5 (11.4)	48.0 (12.5)	51.2 (11.4)
Years of formal education				
Less than 10 years	542 (0.9%)	471 (2.6%)	802 (1.5%)	781 (3.7%)
10 years	19,680 (33.5%)	7463 (41.7%)	22,793 (42.8%)	10,651 (49.9%)
More than 10 years	38,472 (65.5%)	9966 (55.7%)	29,615 (55.7%)	9907 (46.4%)
Migration background (yes)	7532 (12.8%)	3126 (17.5%)	6991 (13.1%)	3478 (16.3%)
CTS sum score	6.17 (1.23)	10.47 (2.49)	5.97 (1.18)	10.67 (3.11)
Number of CM subtypes	–	1.43 (0.76)	–	1.59 (0.94)
1	–	12,565 (70.2%)	–	13,655 (64.0%)
2	–	3500 (19.6%)	–	4353 (20.4%)
3 or more	–	1835 (10.3%)	–	3331 (15.6%)
Emotional neglect	–	5005 (28.0%)	–	6269 (29.4%)
Physical neglect	–	7563 (42.3%)	–	7411 (34.7%)
Emotional abuse	–	4730 (26.4%)	–	7372 (34.5%)
Physical abuse	–	6382 (35.7%)	–	5772 (27.0%)
Sexual abuse	–	1941 (10.8%)	–	7164 (33.6%)
Body height in cm	179 (7)	178 (7)	166 (6)	165 (7)
Body weight in kg	87 (15)	88 (16)	71 (15)	72 (16)
BMI in kg/m^2^	27.0 (4.4)	27.7 (4.6)	25.7 (5.3)	26.5 (5.7)
Waist circumference in cm	96 (13)	98 (13)	85 (13)	87 (14)
Increased waist circumference	16,294 (27.8%)	6135 (34.3%)	18,272 (34.3%)	8768 (41.1%)
Waist to height ratio in %	53.5 (7.3)	55.2 (7.5)	51.1 (8.2)	52.7 (8.7)
Fat mass in %	25.2 (7.0)	26.5 (7.0)	35.6 (7.8)	37.0 (7.7)
Subcutaneous adipose tissue in mm	20.2 (8.2)	20.2 (8.1)	22.3 (10.4)	23.1 (10.2)
Visceral adipose tissue in mm	72.7 (23.7)	76.7 (24.6)	53.1 (20.3)	56.4 (21.5)

Data are expressed as mean and standard deviation for continuous data or as absolute numbers and percentages for categorical data. Percentages refer to the relative proportion of the total number of participants for each sex.

*BMI* body mass index, *CM* childhood maltreatment, *CM−* no CM subtype with moderate to severe exposure, *CM+* ≥1 CM subtype with moderate to severe exposure, *CTS* Childhood Trauma Screener.

### Associations of the CTS sum score with anthropometric and body fat markers

In males, a one-point increase in the CTS sum score was associated with a 0.08 cm lower body height after adjustment for age, years of formal education, and examination center (Table 2). For all other anthropometric and fat markers except VAT, associations of the CTS sum score were stronger in females than in males (Table 2). In females, a one-point increase in the CTS sum score was associated with a 0.08 kg/m^2^ higher BMI (0.05 kg/m^2^ in males), a 0.21 cm larger waist circumference (0.14 cm in men), and a 0.10 mm higher SAT (−0.01 mm in males). The association between the CTS sum score and VAT was similar in men (0.31 mm per one-point increase) and females (0.28 mm per one-point increase). Additional adjustment for migration background did not substantially alter the observed associations (Table S1; Supplementary), except for body weight, where the CTS sum score was no longer significantly associated in females. Positive associations of CTS sum scores with overweight, obesity and high waist circumference were comparable in magnitude between males and females (Table 3). Explorative analyses for age-dependent associations revealed that association strengths for CTS sum scores and anthropometric (Fig. S2) and body fat markers (Fig. S3) generally decline with increasing age. After Bonferroni adjustment, CTS sum score x age interaction terms were significant for all outcomes in male participants, while this was only true for weight and BMI in female participants.

**Table 2:** Sex-stratified associations of total and cumulative childhood maltreatment exposure with anthropometric and body fat markers.

Outcome	CTS sum score	1 vs. no CM	2 vs. no CM	3+ vs. no CM
Body height in cm				
Males	−0.08 (−0.10; −0.06)^a^	−0.39 (−0.52; −0.25)^a^	−0.38 (−0.61; −0.15)^a^	−0.69 (−1.01; −0.37)^a^
Females	−0.03 (−0.05; −0.01)^a^	−0.05 (−0.17; 0.07)	−0.38 (−0.58; −0.19)^a^	−0.15 (−0.37; 0.07)
Body weight in kg				
Males	0.10 (0.05; 0.14)^a^	0.26 (−0.03; 0.55)	0.93 (0.42; 1.45)^a^	1.46 (0.76; 2.16)^a^
Females	0.20 (0.16; 0.24)^a^	0.52 (0.24; 0.80)^a^	1.42 (0.96; 1.88)^a^	2.90 (2.38; 3.43)^a^
Body mass index in kg/m^2^				
Males	0.05 (0.04; 0.07)^a^	0.20 (0.11; 0.28)^a^	0.38 (0.24; 0.53)^a^	0.66 (0.47; 0.86)^a^
Females	0.08 (0.07; 0.10)^a^	0.19 (0.09; 0.29)^a^	0.63 (0.47; 0.79)^a^	1.12 (0.93; 1.30)^a^
Waist circumference in cm				
Males	0.14 (0.10; 0.17)^a^	0.38 (0.15; 0.60)^a^	1.09 (0.69; 1.49)^a^	1.62 (1.07; 2.17)^a^
Females	0.21 (0.18; 0.25)^a^	0.53 (0.29; 0.77)^a^	1.65 (1.26; 2.04)^a^	2.73 (2.29; 3.17)^a^
Waist to height ratio in %				
Males	0.10 (0.08; 0.12)^a^	0.31 (0.18; 0.44)^a^	0.71 (0.49; 0.94)^a^	1.10 (0.80; 1.41)^a^
Females	0.14 (0.12; 0.16)^a^	0.33 (0.18; 0.48)^a^	1.11 (0.87; 1.35)^a^	1.68 (1.41; 1.95)^a^
Relative fat mass in %				
Males	0.04 (0.02; 0.05)^a^	0.14 (0.02; 0.27)	0.31 (0.08; 0.54)	0.65 (0.35; 0.96)^a^
Females	0.07 (0.05; 0.09)^a^	0.12 (−0.01; 0.26)	0.58 (0.36; 0.80)^a^	1.16 (0.91; 1.41)^a^
Subcutaneous adipose tissue in mm				
Males	−0.01 (−0.06; 0.04)	0.08 (−0.23; 0.39)	−0.35 (−0.92; 0.22)	0.13 (−0.67; 0.94)
Females	0.10 (0.05; 0.16)^a^	0.33 (−0.07; 0.72)	0.81 (0.14; 1.48)	1.31 (0.53; 2.08)^a^
Visceral adipose tissue in mm				
Males	0.31 (0.18; 0.45)^a^	1.28 (0.42; 2.13)	1.34 (−0.22; 2.89)	3.17 (0.97; 5.37)
Females	0.28 (0.17; 0.39)^a^	0.94 (0.17; 1.70)	2.65 (1.35; 3.96)^a^	2.58 (1.07; 4.09)^a^

Data are reported as *β* coefficients and 95% confidence intervals derived from sex-stratified linear regression models adjusted for age, years of formal education, and examination center. CTS sum scores exhibit larger average positive associations with most obesity-related outcomes in females as opposed to males. Cumulative CM exposure is associated with weight, BMI, waist circumference, and relative fat mass in a dose-response manner. Average dose-response associations are larger in females compared to males. Only height exhibits negative associations with CM and more consistently so in males as opposed to females.

*CM* childhood maltreatment, *CTS* Childhood Trauma Screener.

^a^Significant after Bonferroni correction (*p* < 0.003).

**Table 3:** Sex-stratified associations of total and cumulative childhood maltreatment exposure with overweight, obesity, and high waist circumference.

Outcome	CTS sum score	1 vs. no CM	2 vs. no CM	3+ vs. no CM
Overweight^a^				
Males	1.01 (1.00; 1.02)*	1.04 (1.00; 1.09)	1.10 (1.01; 1.20)	1.17 (1.04; 1.31)
Females	1.01 (1.01; 1.02)*	1.05 (1.00; 1.09)	1.10 (1.02; 1.19)	1.17 (1.07; 1.27)*
Obesity^b^				
Males	1.03 (1.03; 1.04)*	1.12 (1.06; 1.18)*	1.32 (1.19; 1.45)*	1.51 (1.32; 1.72)*
Females	1.03 (1.03; 1.04)*	1.09 (1.03; 1.15)*	1.30 (1.19; 1.41)*	1.56 (1.43; 1.71)*
High waist circumference^c^				
Males	1.02 (1.02; 1.03)*	1.05 (1.00; 1.10)	1.22 (1.13; 1.31)*	1.31 (1.18; 1.44)*
Females	1.03 (1.02; 1.03)*	1.06 (1.02; 1.11)	1.24 (1.16; 1.32)*	1.39 (1.29; 1.50)*

Data are reported as odds ratios (95% confidence intervals) for BMI-based outcomes (overweight and obesity) and high waist-circumference derived from sex-stratified logistic regression models. All models are adjusted for age, years of formal education, and examination center. Cumulative CM exposure is associated with overweight, obesity, and high waist circumference in a dose-response manner. Odds ratios for obesity and high waist circumference are comparable across exposure categories between females and males, except in individuals with high exposure (i.e., ≥3 CM subtypes), where females exhibit higher odds ratios. This pattern was not observed when overweight was the outcome, as odds ratios were comparable across exposure categories and sex.

*CI* confidence interval, *CM* childhood maltreatment, *CTS* Childhood Trauma Screener, *OR* odds ratio.

^a^Overweight is defined as ≥25 kg/m^2^ BMI < 30 kg/m^2^.

^b^Obesity is defined as BMI ≥ 30 kg/m^2^.

^c^Increased waist circumferences are defined as ≥88 cm for females and ≥102 cm for males.

*Significant after Bonferroni correction (*p* < 0.003).

### Associations of cumulative CM exposure with anthropometric and body fat markers

**Fig. 1:**
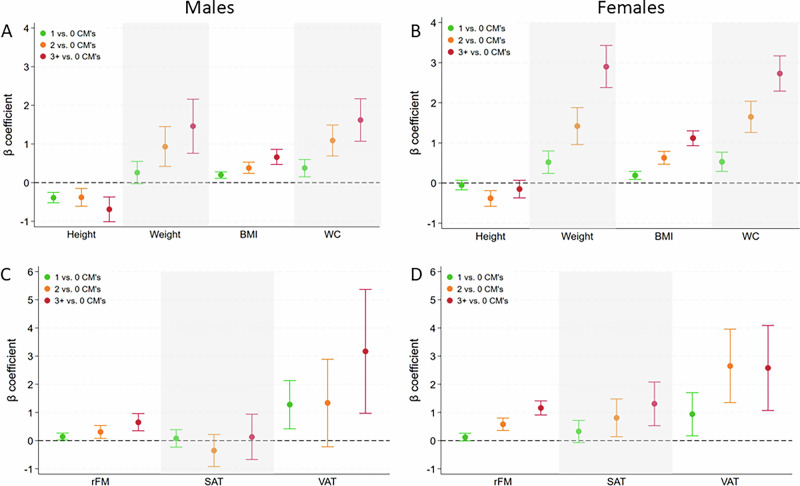
Sex-stratified associations between cumulative exposure to childhood maltreatment and anthropometric and body fat markers. Childhood maltreatment and anthropometric (**A**, **B**) and body fat (**C**, **D**) markers. The left column shows results for males (**A**, **C**); the right column shows results for females (**B**, **D**). Boxplots visualize the sex-specific associations indicated as standardized β-coefficients and 95% confidence intervals*. Colors indicate exposure to one (green), two (yellow), or three or more (red) CM subtypes of at least moderate severity. Cumulative CM exposure is associated with weight, BMI, waist circumference, and relative fat mass in a dose-response manner. Average association strengths are larger in females compared to males. Only height exhibits negative associations with CM and more consistently so in men as opposed to females. Note: *β-coefficients and 95% confidence intervals are derived from sex-disaggregated linear regression models adjusted for age, years of formal education, and study center. BMI body mass index, CM child maltreatment, CTS Childhood Trauma Screener, rFM relative fat mass, SAT subcutaneous adipose tissue, VAT visceral adipose tissue.

In both sexes, most anthropometric measures increased with exposure to multiple CM subtypes in a dose-response relationship, while effects were consistently larger in females compared to males (Table 2 and Fig. 1). Only body height was inversely associated with cumulative exposure to CM categories, especially in males. Compared to males without any CM, males with one CM had on average a 0.39 cm lower body height, while males with three or more CM subtypes had on average a 0.69 cm lower body height. Compared to females without any CM, females with one CM had on average a 0.19 kg/m^2^ higher BMI, a 0.53 cm higher waist circumference, whereas females with three or more CM subtypes had on average a 1.12 kg/m^2^ higher BMI and a 2.73 cm higher waist circumference, and a 1.31 mm higher SAT.

**Fig. 2:**
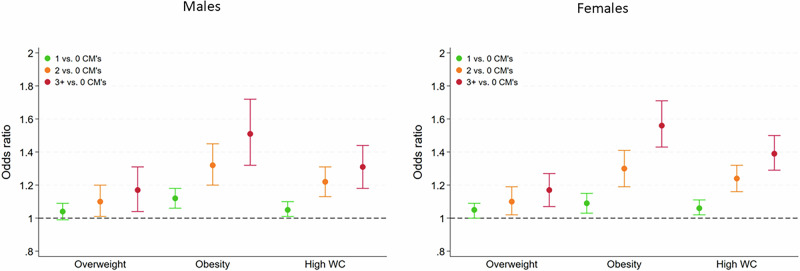
Sex-stratified associations between the cumulative exposure to childhood maltreatment and overweight, obesity, and high waist circumference. The left column shows results for males; the right column shows results for females. Boxplots visualize the sex-specific associations indicated as odds ratios and 95% confidence intervals*. Colors indicate exposure to one (green), two (yellow), or three or more (red) CM subtypes of at least moderate severity. Cumulative CM exposure is associated with overweight, obesity, and high waist circumference in a dose-response manner. Odds ratios for obesity and high waist circumference are comparable across exposure categories between females and males, except in individuals with high exposure (i.e., ≥3 CM subtypes), where females exhibit higher odds ratios. This pattern was not observed when overweight was the outcome, as odds ratios were comparable across exposure categories in both sexes. * Odds ratios and 95% confidence intervals are derived from sex-disaggregated logistic regression models adjusted for age, years of formal education, and study center. Overweight is defined as 25 kg/m^2^ ≥ BMI < 30 kg/m^2^, obesity is defined as BMI ≥ 30 kg/m^2^, and high waist circumferences are defined as ≥88 cm for females and ≥102 cm for males. CM child maltreatment, WC waist circumference.

Moreover, cumulative CM exposure was associated with overweight, general (i.e., BMI ≥ 30 kg/m^2^) and central (i.e., high WC) obesity in a dose-response relationship (see Table 3 and Fig. 2). Associations with overweight were comparable between males and females. Notably, odds ratios for obesity and high waist circumference were comparable across exposure categories between females and males, except in individuals with high exposure (i.e., ≥3 CM subtypes), where females exhibit higher odds ratios (see Table 3 and Fig. 2).

Positive associations between cumulative CM exposure and body fat markers were primarily seen in females with exposure to at least two CM categories. After Bonferroni correction, males with exposure to at least three types of CM exhibited 0.65% increased relative fat mass compared to unexposed males. Females with exposure to at least 2 types of CM had higher relative fat mass and higher VAT compared to unexposed females. Significantly increased SAT was only seen in females with exposure to three or more CM categories. All results are presented in Table 2 and Fig. 1. Additional adjustment for migration background (Table S1) left association patterns essentially unchanged, with the exception of body height in males with exposure to two CM categories, which was no longer significant (*β* = −0.15; 95% CI: −0.39; 0.08).

### Associations of CM subtypes with anthropometric and body fat markers

Associations of CM subtypes and obesity-related traits are displayed in Table S2. In both sexes, emotional and physical abuse exhibited the most consistent and largest effects on obesity-related traits. Additionally, emotional neglect and sexual abuse were significantly associated with most outcomes in females, but not in males. In males and females alike, physical neglect showed the strongest inverse association with body height. Additional adjustment for migration background did not alter association patterns significantly (Table S3).

## Discussion

The purpose of the present study was to conceptually replicate and extend our previously published data [12] on sex-specific associations between CM exposure and obesity-related traits in the general adult population. The patterns of association can be summarized as follows: (1) the association of CM exposure and obesity-related traits is generally stronger in females than in males; (2) in both sexes, exposure to multiple CM subtypes exhibits a dose-response relationship with most obesity-related traits; (3) associations between CM exposure and obesity indicators (i.e., BMI ≥ 30 kg/m^2^ and high waist circumference) were stronger than associations with overweight, (4) in both sexes and across obesity-related traits, physical and emotional abuse emerge as the exposures with the strongest average associations and are associated with the most outcomes; (5) emotional neglect and sexual abuse exhibit significant associations with most obesity-related traits in females, but not males. Finally, explorative analyses revealed an overall age-dependent decline in association strength between CM exposure (CTS sum score) and anthropometric and body fat markers, which was more pronounced in male compared to female participants.

Overall, results are consistent with previous meta-analytical and population-based findings [7, 12, 13, 22, 23] that have shown stronger associations between CM exposure and obesity-related traits in females compared to males, strengthening confidence in this pattern of associations. Concerning the dose-response relationship between CM exposure and most obesity-related traits observed in both males and females, our findings are further in line with the results of the original *Adverse Childhood Experiences (ACE)* study [4]. Among other health outcomes, Felitti et al. [4] demonstrated, without reporting sex-disaggregated results, that with increasing numbers of ACEs, participants had an increased risk of severe obesity (i.e., BMI ≥ 35 kg/m^2^) compared to unexposed individuals. Our results indicate that this association is comparable in effect size already at a lower threshold for obesity (i.e., BMI ≥ 30 kg/m^2^). Hughes et al. [6] in their meta-analysis reported a combined OR for the association of ACEs with overweight and obesity of 1.39. We here report that the magnitude of association depends on the outcome and is larger in models with obesity as the outcome (Table 3). Notably, sex differences in association strength emerge only for obesity and high waist circumference, but not for overweight at high levels of CM exposure (i.e., ≥3 CM subtypes), where females exhibit higher odds ratios (see Table 3 and Fig. 2). Of note and unlike in our previous study [12], most associations between CM exposure and obesity-related traits were also significant in male participants, which is likely due to the substantially increased sample size (SHIP study: *n* = 4006 vs. NAKO study: *n* = 151,143) and the resulting greater statistical power to detect associations of smaller magnitude.

The finding that the CTS sum score is related to comparable increases in VAT in males and females alike, but more consistently across cumulative and specific types of CM exposure in females, may be of particular interest. One hallmark of the sexual dimorphism in adult body composition is that compared to premenopausal females, males accrue more VAT at comparable BMI [15, 16]. This male-type body fat distribution is associated with greater cardiometabolic risk, like insulin resistance, hypertension, dyslipidemia, and chronic inflammation [24, 25]. These VAT-associated alterations may partly underlie obesity-related complications in survivors of CM. Interestingly, females with exposure to CM exhibit a significant accumulation of VAT, which may indicate a tendency of these females to develop an “*android*” adult phenotype of body fat distribution, which may predispose these females to increased cardiometabolic health risks. In this context, exploratory analyses also showed age-dependent associations between CM exposure and obesity-related traits with some notable sex differences seen especially for body fat markers. While in male participants, associations between CTS sum scores and body fat markers declined with increasing age in our cohort, this was not observed in females. This may suggest a prolonged exposure to VAT-related physiological alterations in CM-exposed females compared to males, adding a potential additional source by which CM exposure may contribute to sex differences in long-term health outcomes. These hypotheses should be considered with precaution, however, as it needs further validation and a better understanding of the underlying linking mechanisms. Being aware of the multifactorial etiology of increased VAT, we speculate that, especially in female subjects, estrogen signaling may be a promising candidate mechanism for future research. Estrogens have pleiotropic effects with regard to fat metabolism and distribution [26], energy expenditure [27], and central control of food intake [28]. Moreover, estrogen deficiency as seen in postmenopausal females induces a shift towards higher relative amounts of VAT and decreased energy expenditure [27]. The role of this estrogen pathway in CM-associated increases in VAT in females is unclear, however, due to a lack of research. Animal models have established that early postnatal stress predicts reduced expression of estrogen-receptor α (ERα) in hypothalamic neurons of female offspring [29]. Given that ERα-mediated signaling is critical for estrogen effects in both behavioral and physiological mechanisms related to obesity and body fat distribution [26, 28], this pathway could represent a plausible target mechanism to better understand the increased levels of VAT in females after CM exposure.

It is also important to note that the cardiometabolic correlates of CM exposure in males should not be underestimated either and may differ from those seen in females. For example, we have previously shown that only in males but not in females CM exposure is associated with increased liver fat content and increased odds for metabolic dysfunction-associated steatotic liver disease [30], suggesting an alternate pathway through which CM exposure may confer increased cardiometabolic risk in males.

An exception from the general pattern of stronger associations observed in females represents body height, for which CM showed a stronger association in males, which we have also shown before [12]. Body growth is a sexually dimorphic and dynamic process that occurs during a relatively long period, i.e., from fetal life until late puberty/early adulthood [31]. During this extended developmental time window, individuals are particularly prone to the deleterious long-term effects of chronic stress, such as CM [32, 33]. In addition, body height is strongly regulated by genetic and endocrine mechanisms, which may interact with or be responsive to severe stressors occurring during the early stages of life in an age- and sex-dependent manner to account for individual differences in body height [31]. Previous research has shown that in two independent birth cohorts, CM exposure in the form of either neglect at 7 years [34] or abuse and neglect between 0 and 14 years [35] was prospectively associated with modest but significant reductions in adult height, regardless of sex. Moreover, early life conditions that often co-occur with CM, such as low childhood socioeconomic status (SES), have also been linked to reduced body growth [36]. In this regard, it is worth noting that the strongest associations with adult body height were observed for childhood physical neglect in both males and females, an exposure that may be indicative of low childhood SES. We have not measured childhood SES and caution is therefore warranted to solely attribute the effects of childhood physical neglect on adult body height (and other health outcomes) to neglectful parenting. They may also represent health effects associated with conditions that occur more frequently in low SES contexts, such as malnutrition.

Several limitations need to be considered. First, the method of assessing CM exposure poses several challenges. Participants provided retrospective self-reports about their CM exposure using the CTS, a 5-item screening instrument. Each CM subtype is represented by only one item. However, the CTS has demonstrated good agreement with the Childhood Trauma Questionnaire (CTQ) [19] and can be considered a reliable short version of the CTQ, which is a well-established instrument in CM-related research. Furthermore, retrospective self-report questionnaires such as the CTS may be influenced in several ways, e.g., through recollection errors, forgetting, exaggeration, or denial [37]. However, a previous meta-analysis has shown that CM-obesity associations were comparable between studies that used retrospective and prospective measures of CM exposure [7].

Second, other dimensions of diversity, such as age, SES, or race/ethnicity, may also contribute to differential obesity-related cardiometabolic risk after CM. We have conducted our analyses in an ethnically relatively homogenous white German population, while past research has shown that the CM-obesity association may be stronger in samples with higher relative proportions of white participants [7]. This circumstance may thus decrease the generalizability of the current study to populations of more diverse ethnic backgrounds and warrants further study. However, supplemental analyses including migration background as an additional covariate did not alter the overall pattern of associations. With regard to a possible effect modification by age in the CM-obesity association, a longitudinal study in adults [38] found consistently elevated WC over a 9-year follow-up in CM-exposed individuals, but no evidence for a CM x time interaction.

Third, the response rate to participate in the NAKO study was relatively low at 17% [17] with substantial variation between study centers (9–32%). This may introduce biases related to sample collection (e.g., selection bias). Therefore, the NAKO study population cannot be considered representative for the German general population and basic characteristics of the sample that are relevant to the present study, such as CM prevalence are likely underestimated.

Fourth, we considered biological sex as an analytical variable in our study, while not (or only indirectly) addressing the role of gender, which among others captures aspects of gender identity, gender roles, and power hierarchies associated with gender. As an example, and consistent with prior research [39], we found that among individuals with CM exposure, females are approximately three times more likely than males to report childhood sexual abuse. A pathway towards prevention of this pernicious form of violence, which disproportionately affects female individuals needs to adopt gender rather than sex sensitive perspective to accurately address this phenomenon.

As another limitation, we have conducted cross-sectional analyses and could therefore not establish longitudinal or causal associations. In this regard, it is important to note that the effects of CM on obesity are already seen in children and adolescents, particularly in girls [40, 41], indicating that biological embedding (e.g., through epigenetic mechanisms; altered stress physiology) and behavioral alterations (e.g., disordered eating; physical inactivity) related to obesity may manifest early after CM exposure in a sex-specific manner. Adding an additional level of complexity to this body of evidence, Moog et al. [42] recently reported that maternal exposure to CM predicts obesity in their female but not male children, suggesting an intergenerational mother-to-daughter transmission of CM-associated obesity. Together, these results indicate that CM exposure, both intra- and intergenerationally, is associated with early onset obesity in girls, which may in turn expose these female individuals to prolonged periods of obesity-related alterations in metabolic homeostasis and increased cardiovascular risk (e.g., increased inflammation), possibly contributing to the premature mortality seen in adult females (but not males) after CM exposure [10].

It has also been shown that variables related to CM, such as age at exposure may contribute to differences in CM-associated long-term health outcomes [43]. We suggest that future research on CM-related long-term health outcomes should more comprehensively characterize characteristics of the exposure beyond the different types of maltreatment. As another limitation, we should note the absence of genetic risk scores in our study. Obesity has a considerable genetic component [44]. Therefore, future studies exploring associations between CM exposure and obesity should include genetic risk scores (i.e., gene-environment interactions) to further refine our understanding of CM-related obesity risk.

Finally, we acknowledge that we have not tested potential mediators in the present study. In this regard, several current reviews have provided narrative [45] and quantitative [46] syntheses on the various biological and behavioral pathways that may partly explain the association of CM exposure and obesity-related traits in adulthood. We propose to further investigate the role of depression in the context of sex-specific CM-obesity associations, given its particular clinical relevance in this context. CM is an established risk factor for depressive disorders [47, 48] that are characterized by higher recurrence risk, treatment resistance [49], and comorbid cardiometabolic disease [50]. Simultaneously, females are more likely than males to be diagnosed with depression after CM [5] and we have recently shown that recurrent major depression, which is more likely after CM exposure [49], is associated with increased obesity-related traits in females, but not in males [51]. As a next step, we propose that future research, by focusing on sex differences, should try to integrate these findings in order to identify subgroups of individuals with CM exposure, more severe (e.g., recurrent and/or treatment resistant) forms of depression, and concurrent obesity-related cardiometabolic risk, which may help to tailor individualized treatments within a framework of personalized medicine.

A major strength of our study is the sex-stratified analytical approach that takes into consideration the sexual dimorphism in adult obesity [16] and the sex-specific pattern of exposure to different CM subtypes [52]. Moreover, the large sample size of 151,143 individuals provides robust estimates of CM-associated alterations in adult obesity-related traits, and the fact that, in addition to standard anthropometric markers, we also obtained direct measures of body fat through BIA and ultrasound. Body fat tissue, and especially VAT, is associated with increased cardiometabolic risk in people with obesity [24], and most pertinent studies have considered BMI or waist circumference as the primary outcomes in association with CM exposure [7, 53]. The present study may therefore provide a valuable contribution to stimulate further research into the sex-specific pathways that mediate the CM-obesity association in order to achieve a more accurate prediction of chronic obesity-related complications in both males and females after CM.

Taken together and bearing the limitations mentioned above in mind, our data suggest a nuanced pattern of associations between CM exposure and anthropometric and body fat markers that may depend on the sex of the exposed individual, the cumulative severity and type of CM, as well as the measured outcome. In light of the rapidly increasing levels of obesity and the consistently high rates of child maltreatment worldwide, it appears imperative to implement early sex-and gender sensitive prevention strategies to either reduce the exposure to CM or mitigate their detrimental long-term effects on health.

## Supplementary information

Supplemental Material

## Data Availability

NAKO data can be obtained via an electronic application portal (https://transfer.nako.de).

## References

[1] Grummitt LR, Kreski NT, Kim SG, Platt J, Keyes KM, McLaughlin KA. Association of childhood adversity with morbidity and mortality in US adults: a systematic review. JAMA Pediatr. 2021;175:1269–78. 34605870 10.1001/jamapediatrics.2021.2320 PMC9059254

[2] Dong M, Giles WH, Felitti VJ, Dube SR, Williams JE, Chapman DP, et al. Insights into causal pathways for ischemic heart disease: adverse childhood experiences study. Circulation. 2004;110:1761–6. 15381652 10.1161/01.CIR.0000143074.54995.7F

[3] Merrick MT. Vital signs: estimated proportion of adult health problems attributable to adverse childhood experiences and implications for prevention—25 states, 2015–2017. Morb Mortal Wkly Rep. 2019;68:999–1005. 10.15585/mmwr.mm6844e1 PMC6837472 31697656

[4] Felitti VJ, Anda RF, Nordenberg D, Williamson DF, Spitz AM, Edwards V, et al. Relationship of childhood abuse and household dysfunction to many of the leading causes of death in adults: the Adverse Childhood Experiences (ACE) Study. Am J Prev Med. 1998;14:245–58. 9635069 10.1016/s0749-3797(98)00017-8

[5] Klinger-König J, Erhardt A, Streit F, Völker MP, Schulze MB, Keil T, et al. Childhood trauma and somatic and mental illness in adulthood: findings of the NAKO Health Study. Dtsch Arztebl Int. 2024;121:1. 37876295 10.3238/arztebl.m2023.0225 PMC10916765

[6] Hughes K, Bellis MA, Hardcastle KA, Sethi D, Butchart A, Mikton C, et al. The effect of multiple adverse childhood experiences on health: a systematic review and meta-analysis. Lancet Public Health. 2017;2:e356–66. 29253477 10.1016/S2468-2667(17)30118-4

[7] Danese A, Tan M. Childhood maltreatment and obesity: systematic review and meta-analysis. Mol Psychiatry. 2014;19:544–54. 23689533 10.1038/mp.2013.54

[8] World Health Organization. Obesity and overweight. 2021. https://www.who.int/news-room/fact-sheets/detail/obesity-and-overweight.

[9] GBD. Health effects of overweight and obesity in 195 countries over 25 years. N Engl J Med. 2017;377:13–27. 28604169 10.1056/NEJMoa1614362 PMC5477817

[10] Chen E, Turiano NA, Mroczek DK, Miller GE. Association of reports of childhood abuse and all-cause mortality rates in women. JAMA Psychiatry. 2016;73:920–7. 27540997 10.1001/jamapsychiatry.2016.1786 PMC5234580

[11] Mauvais-Jarvis F, Merz NB, Barnes PJ, Brinton RD, Carrero J-J, DeMeo DL, et al. Sex and gender: modifiers of health, disease, and medicine. Lancet. 2020;396:565–82. 32828189 10.1016/S0140-6736(20)31561-0 PMC7440877

[12] Töpfer P, Siewert-Markus U, Klinger-König J, Grabe HJ, Stracke S, Dörr M, et al. Sex-specific associations of childhood maltreatment with obesity-related traits-the Study of Health in Pomerania (SHIP). Child Abus Negl. 2024;149:106704. 10.1016/j.chiabu.2024.106704 38395019

[13] Fuller-Thomson E, Sinclair DA, Brennenstuhl S. Carrying the pain of abuse: gender-specific findings on the relationship between childhood physical abuse and obesity in adulthood. Obes Facts. 2013;6:325–36. 23970142 10.1159/000354609 PMC5644736

[14] Beltz AM, Beery AK, Becker JB. Analysis of sex differences in pre-clinical and clinical data sets. Neuropsychopharmacology. 2019;44:2155–8. 31527863 10.1038/s41386-019-0524-3 PMC6898365

[15] Power ML, Schulkin J. Sex differences in fat storage, fat metabolism, and the health risks from obesity: possible evolutionary origins. Br J Nutr. 2008;99:931–40. 17977473 10.1017/S0007114507853347

[16] Palmer BF, Clegg DJ. The sexual dimorphism of obesity. Mol Cell Endocrinol. 2015;402:113–9. 25578600 10.1016/j.mce.2014.11.029 PMC4326001

[17] Peters A, German National Cohort C, Peters A, Greiser KH, Gottlicher S, Ahrens W, et al. Framework and baseline examination of the German National Cohort (NAKO). Eur J Epidemiol. 2022;37:1107–24. 36260190 10.1007/s10654-022-00890-5 PMC9581448

[18] Grabe HJ, Schulz A, Schmidt CO, Appel K, Driessen M, Wingenfeld K, et al. [A brief instrument for the assessment of childhood abuse and neglect: the childhood trauma screener (CTS)]. Psychiatr Prax. 2012;39:109–15. 22422160 10.1055/s-0031-1298984

[19] Glaesmer H, Schulz A, Hauser W, Freyberger HJ, Brahler E, Grabe HJ. [The childhood trauma screener (CTS)—development and validation of cut-off-scores for classificatory diagnostics]. Psychiatr Prax.2013;40:220–6. 23564353 10.1055/s-0033-1343116

[20] Fischer B, Sedlmeier AM, Hartwig S, Schlett CL, Ahrens W, Bamberg F. et al. [Anthropometric measures in the German National Cohort-more than weight and height].Bundesgesundheitsblatt Gesundheitsforschung Gesundheitsschutz. 2020;63:290–300. 32020361 10.1007/s00103-020-03096-w

[21] Wiessner C, Keil T, Krist L, Zeeb H, Dragano N, Schmidt B, et al. Personen mit Migrationshintergrund in der NAKO Gesundheitsstudie–soziodemografische Merkmale und Vergleiche mit der autochthonen deutschen Bevölkerung. 2020. 10.1007/s00103-020-03097-9 32034443

[22] Bernstein CN, Fisk JD, Walld R, Bolton JM, Sareen J, Patten SB, et al. Use of benzodiazepines and Z-drugs in inflammatory Bowel disease. Am J Gastroenterol. 2022;117:2046–54. 36288107 10.14309/ajg.0000000000001955

[23] Ernst M, Tibubos AN, Werner A, Beutel ME, Plener PL, Fegert JM, et al. Sex-dependent associations of childhood neglect and bodyweight across the life span. Sci Rep. 2019;9:5080. 30911019 10.1038/s41598-019-41367-y PMC6434018

[24] Ibrahim MM. Subcutaneous and visceral adipose tissue: structural and functional differences. Obes Rev. 2010;11:11–8. 19656312 10.1111/j.1467-789X.2009.00623.x

[25] Wajchenberg BL. Subcutaneous and visceral adipose tissue: their relation to the metabolic syndrome. Endocr Rev. 2000;21:697–738. 11133069 10.1210/edrv.21.6.0415

[26] Leeners B, Geary N, Tobler PN, Asarian L. Ovarian hormones and obesity. Hum Reprod Update. 2017;23:300–21. 28333235 10.1093/humupd/dmw045 PMC5850121

[27] Lovejoy JC, Champagne C, De Jonge L, Xie H, Smith S. Increased visceral fat and decreased energy expenditure during the menopausal transition. Int J Obes. 2008;32:949–58. 10.1038/ijo.2008.25 PMC2748330 18332882

[28] Brown LM, Gent L, Davis K, Clegg DJ. Metabolic impact of sex hormones on obesity. Brain Res. 2010;1350:77–85. 20441773 10.1016/j.brainres.2010.04.056 PMC2924463

[29] Champagne FA, Weaver IC, Diorio J, Dymov S, Szyf M, Meaney MJ. Maternal care associated with methylation of the estrogen receptor-α1b promoter and estrogen receptor-α expression in the medial preoptic area of female offspring. Endocrinology. 2006;147:2909–15. 16513834 10.1210/en.2005-1119

[30] Siewert-Markus U, Ittermann T, Klinger-König J, Grabe HJ, Stracke S, Völzke H, et al. Childhood maltreatment and risk of metabolic dysfunction-associated steatotic liver disease—evidence of sex-specific associations in the general population. J Psychosom Res. 2024;183:111829. 10.1016/j.jpsychores.2024.111829 38896985

[31] Mousikou M, Kyriakou A, Skordis N. Stress and growth in children and adolescents. Horm Res Paediatr. 2023;96:25–33. 34814153 10.1159/000521074

[32] Danese A, McEwen BS. Adverse childhood experiences, allostasis, allostatic load, and age-related disease. Physiol Behav. 2012;106:29–39. 21888923 10.1016/j.physbeh.2011.08.019

[33] Lupien SJ, McEwen BS, Gunnar MR, Heim C. Effects of stress throughout the lifespan on the brain, behaviour and cognition. Nat Rev Neurosci. 2009;10:434–45. 19401723 10.1038/nrn2639

[34] Denholm R, Power C, Li L. Adverse childhood experiences and child-to-adult height trajectories in the 1958 British birth cohort. Int J Epidemiol. 2013;42:1399–409. 24019423 10.1093/ije/dyt169

[35] Abajobir AA, Kisely S, Williams G, Strathearn L, Najman JM. Height deficit in early adulthood following substantiated childhood maltreatment: a birth cohort study. Child Abus Negl. 2017;64:71–8. 10.1016/j.chiabu.2016.12.010 28039757

[36] Niere O, Spannemann L, Stenzel P, Bogin B, Hermanussen M, Scheffler C. Plasticity of human growth—a systematic review on psychosocial factors influencing growth. Anthropol Anz. 2020;77:431–43. 10.1127/anthranz/2020/1223 32432643

[37] Reuben A, Moffitt TE, Caspi A, Belsky DW, Harrington H, Schroeder F, et al. Lest we forget: comparing retrospective and prospective assessments of adverse childhood experiences in the prediction of adult health. J Child Psychol Psychiatry. 2016;57:1103–12. 27647050 10.1111/jcpp.12621 PMC5234278

[38] Souama C, Milaneschi Y, Lamers F, Vinkers CH, Giltay EJ, Liemburg EJ, et al. Metabolic syndrome after childhood trauma: a 9-year longitudinal analysis. Psychol Med. 2024;54:1373–81. 37981868 10.1017/S0033291723003264

[39] Stoltenborgh M, Van Ijzendoorn MH, Euser EM, Bakermans-Kranenburg MJ. A global perspective on child sexual abuse: meta-analysis of prevalence around the world. Child Maltreat. 2011;16:79–101. 21511741 10.1177/1077559511403920

[40] Schroeder K, Schuler BR, Kobulsky JM, Sarwer DB. The association between adverse childhood experiences and childhood obesity: a systematic review. Obes Rev. 2021;22:e13204. 33506595 10.1111/obr.13204 PMC8192341

[41] Isohookana R, Marttunen M, Hakko H, Riipinen P, Riala K. The impact of adverse childhood experiences on obesity and unhealthy weight control behaviors among adolescents. Compr Psychiatry. 2016;71:17–24. 27580313 10.1016/j.comppsych.2016.08.002

[42] Moog NK, Cummings PD, Jackson KL, Aschner JL, Barrett ES, Bastain TM, et al. Intergenerational transmission of the effects of maternal exposure to childhood maltreatment in the USA: a retrospective cohort study. Lancet Public Health. 2023;8:e226–37. 36841563 10.1016/S2468-2667(23)00025-7 PMC9982823

[43] Klinger-König J, Krause E, Wittfeld K, Friedrich N, Völzke H, Grabe HJ. The age of onset and duration of childhood abuse: an extension of the childhood trauma screener. Child Abus Negl. 2025;163:107354. 10.1016/j.chiabu.2025.107354 40081162

[44] Loos RJ, Yeo GS. The genetics of obesity: from discovery to biology. Nat Rev Genet. 2022;23:120–33. 34556834 10.1038/s41576-021-00414-z PMC8459824

[45] Wallace C, Krugman R. More than what you eat: a review on the association between childhood maltreatment and elevated adult BMI. Curr Nutr Rep. 2024;13:1–5. 10.1007/s13668-024-00558-4 PMC11327177 38922364

[46] Wiss DA, Brewerton TD. Adverse childhood experiences and adult obesity: a systematic review of plausible mechanisms and meta-analysis of cross-sectional studies. Physiol Behav. 2020;223:112964. 32479804 10.1016/j.physbeh.2020.112964

[47] Li M, D’Arcy C, Meng X. Maltreatment in childhood substantially increases the risk of adult depression and anxiety in prospective cohort studies: systematic review, meta-analysis, and proportional attributable fractions. Psychol Med. 2016;46:717–30. 26708271 10.1017/S0033291715002743

[48] Humphreys KL, LeMoult J, Wear JG, Piersiak HA, Lee A, Gotlib IH. Child maltreatment and depression: a meta-analysis of studies using the Childhood Trauma Questionnaire. Child Abus Negl. 2020;102:104361. 10.1016/j.chiabu.2020.104361 PMC7081433 32062423

[49] Nanni V, Uher R, Danese A. Childhood maltreatment predicts unfavorable course of illness and treatment outcome in depression: a meta-analysis. Am J Psychiatry. 2012;169:141–51. 22420036 10.1176/appi.ajp.2011.11020335

[50] Souama C, Lamers F, Milaneschi Y, Vinkers CH, Defina S, Garvert L, et al. Depression, cardiometabolic disease, and their co-occurrence after childhood maltreatment: an individual participant data meta-analysis including over 200,000 participants. BMC Med. 2023;21:93. 36907864 10.1186/s12916-023-02769-y PMC10010035

[51] Bannert U, Siewert-Markus U, Klinger-König J, Grabe HJ, Stracke S, Dörr M, et al. Major depression recurrence is associated with differences in obesity-related traits in women, but not in men. Eur Psychiatry. 2024;67:e55. 10.1192/j.eurpsy.2024.1764 PMC11457113 39301585

[52] Moody G, Cannings-John R, Hood K, Kemp A, Robling M. Establishing the international prevalence of self-reported child maltreatment: a systematic review by maltreatment type and gender. BMC Public Health. 2018;18:1–15. 10.1186/s12889-018-6044-y PMC6180456 30305071

[53] Hemmingsson E, Johansson K, Reynisdottir S. Effects of childhood abuse on adult obesity: a systematic review and meta-analysis. Obes Rev. 2014;15:882–93. 25123205 10.1111/obr.12216

