# Supplementary material for: Sex-dependent associations of childhood maltreatment with obesity-related traits: results from the German National Cohort (NAKO)
Source: Int J Obes (Lond). 2026 Feb 6;50(2):329–37. doi: 10.1038/s41366-025-01914-2 (PMC12913021; doi:10.1038/s41366-025-01914-2)
Supplement: Supplementary file 1 — Supplemental Material [file 41366_2025_1914_MOESM1_ESM.pdf]

Supplement

Sex-dependent Associations of Childhood Maltreatment with Obesity-related Traits: Results from the German National Cohort (NAKO)

Philipp Töpfer[1], Johanna Klinger-König[2], Ulrike Siewert-Markus[2], Sabine Schipf[3], Beate Fischer[4], Anja M. Sedlmeier[4,5,6], Antje Hebestreit[7], Wolfgang Ahrens[7], Klaus Berger[8], Hermann Brenner [9,10,11], Stefanie Do[7], Jana-Kristin Heise[12], Stefanie Jaskulski[13], André Karch[8], Thomas Keil[14,15,16], Carolina Klett-Tammen[12], Michael F. Leitzmann[4], Annette Peters[17,18,19,20], Börge Schmidt[21], Matthias B. Schulze[22,23], Stefan N. Willich[14], Marcus Dörr[24,25], Henry Völzke[3], Marcello RP Markus[24, 25], Sylvia Stracke[1], Hans J. Grabe[2,26], Till Ittermann[3]

Author affiliations

[1] Department of Internal Medicine A; University Medicine Greifswald, Germany

[2] Department of Psychiatry and Psychotherapy; University Medicine Greifswald, Germany

[3] Institute for Community Medicine; Department SHIP Clinical-Epidemiological Research; University Medicine Greifswald, Germany

[4] Department of Epidemiology and Preventive Medicine; University of Regensburg, Germany

[5] Center for Translational Oncology, University Hospital Regensburg, Germany

[6] Bavarian Cancer Research Center (BZKF), Regensburg, Germany

[7] Leibniz Institute for Prevention Research and Epidemiology – BIPS, Bremen, Germany.

[8] Institute of Epidemiology and Social Medicine, University of Münster, Münster, Germany

[9] Division of Clinical Epidemiology and Aging Research, German Cancer Research Center (DKFZ), Heidelberg, Germany

[10] Division of Preventive Oncology, German Cancer Research Center (DKFZ) and National Center for Tumour Diseases (NCT), Heidelberg, Germany

[11] German Cancer Consortium (DKTK), German Cancer Research Center (DKFZ), Heidelberg, Germany

[12] Department of Epidemiology, Helmholtz Center for Infection Research (HZI), Braunschweig, Germany

[13] Institute for Prevention and Cancer Epidemiology, Faculty of Medicine and Medical Center, University of Freiburg, Freiburg, Germany

[14] Institute of Social Medicine, Epidemiology and Health Economics, Charité – University Medicine Berlin, Berlin, Germany

[15] Institute for Clinical Epidemiology and Biometry, University of Würzburg, Würzburg, Germany

[16] State Institute of Health I, Bavarian State Office for Health and Food Safety, Erlangen, Germany
[17] Institute of Epidemiology, Helmholtz Zentrum München - German Research Center for Environmental Health (GmbH), Neuherberg, Germany
[18] Institute for Medical Information Processing, Biometry and Epidemiology, Medical Faculty, Ludwig-Maximilians-Universität München, Munich, Germany
[19] German Center for Diabetes Research (DZD e.V.), Neuherberg, Germany
[20] German Center for Mental Health (DZPG), partner site Munich, Munich, Germany
[21] Institute for Medical Informatics, Biometry and Epidemiology, University Hospital Essen, University Duisburg-Essen, Essen, Germany
[22] Department of Molecular Epidemiology, German Institute of Human Nutrition Potsdam-Rehbruecke, Nuthetal, Germany
[23] Institute of Nutritional Science, University of Potsdam, Nuthetal, Germany
[24] Department of Internal Medicine B, University Medicine Greifswald, Germany
[25] German Centre for Cardiovascular Research (DZHK), partner site Greifswald, Greifswald, Germany.
[26] German Center for Neurodegenerative Diseases (DZNE), Site Rostock/Greifswald, Germany

1. Supplemental Methods

Sample/Sequential Exclusion of Study Participants

For the present analyses, we excluded 32,772 individuals with missing data in the Childhood Trauma Screener (CTS), 6,120 individuals with missing data in the anthropometric markers, and 14,709 individuals with missing data in years of formal education resulting in a study population of 151,143 individuals (74,549 women) aged 19 to 75 years (mean 49.2 years). Data on subcutaneous (SAT) and visceral adipose tissue thickness (VAT) were available in subgroups of 36,111 and 35,719 individuals, respectively. **Figure S1** provides a graphical representation of the sequential exclusion of participants with missing data for study variables.

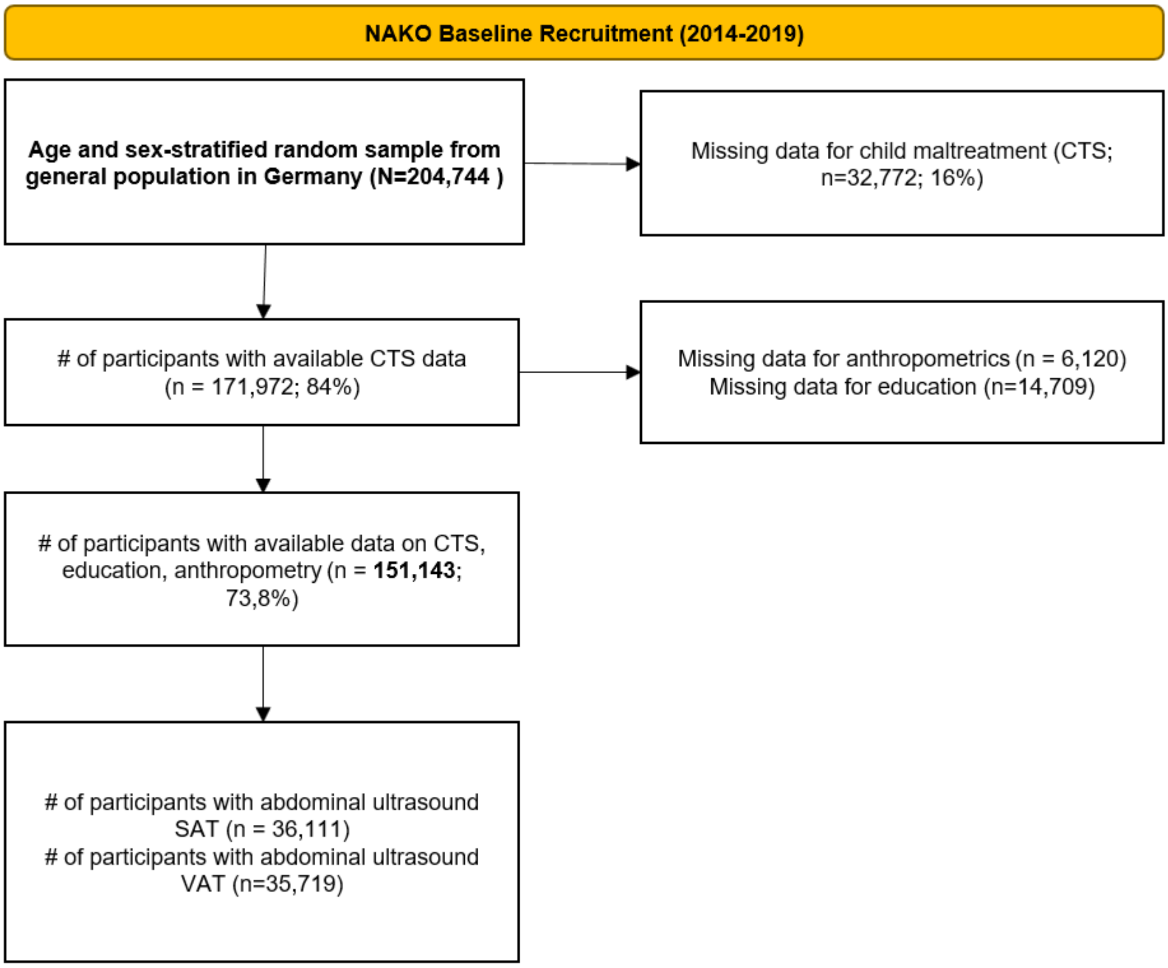

Figure S1: Flow chart of sequential exclusion of subjects with missing data for study variables.

2. Supplemental Results

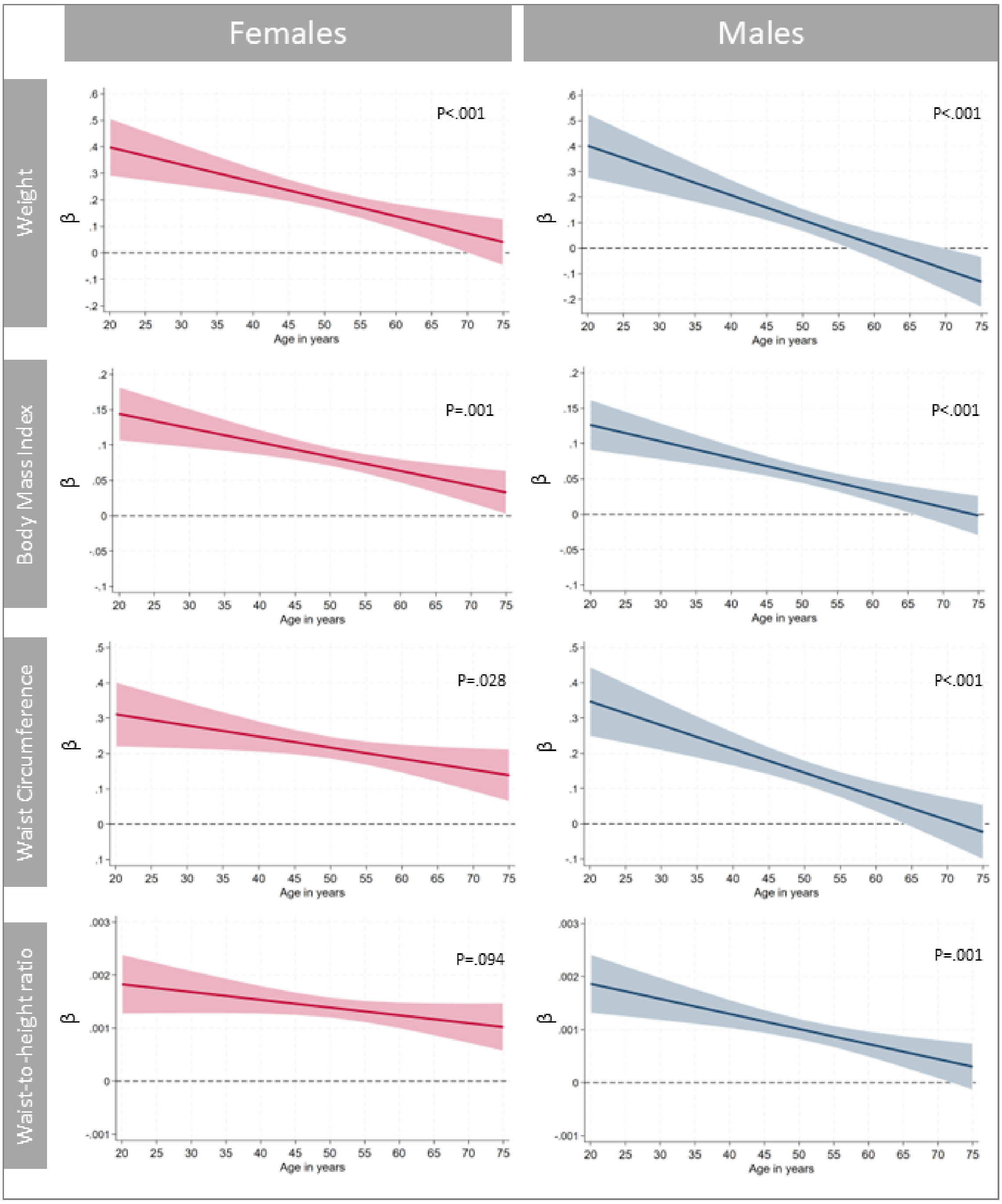

Figure S2: Age-dependent associations between CTS sum score and anthropometric indicators for females (left) and males (right).

Note: Data are shown as β coefficients and 95% confidence intervals (shaded areas) indicating the age-dependent association between CTS sum scores and different anthropometric markers (i.e., weight, body mass index, waist circumference, and waist-to-height ratio) derived from fully adjusted sex-stratified linear regression models.

p-values denote significance levels for the CTS x age interaction.

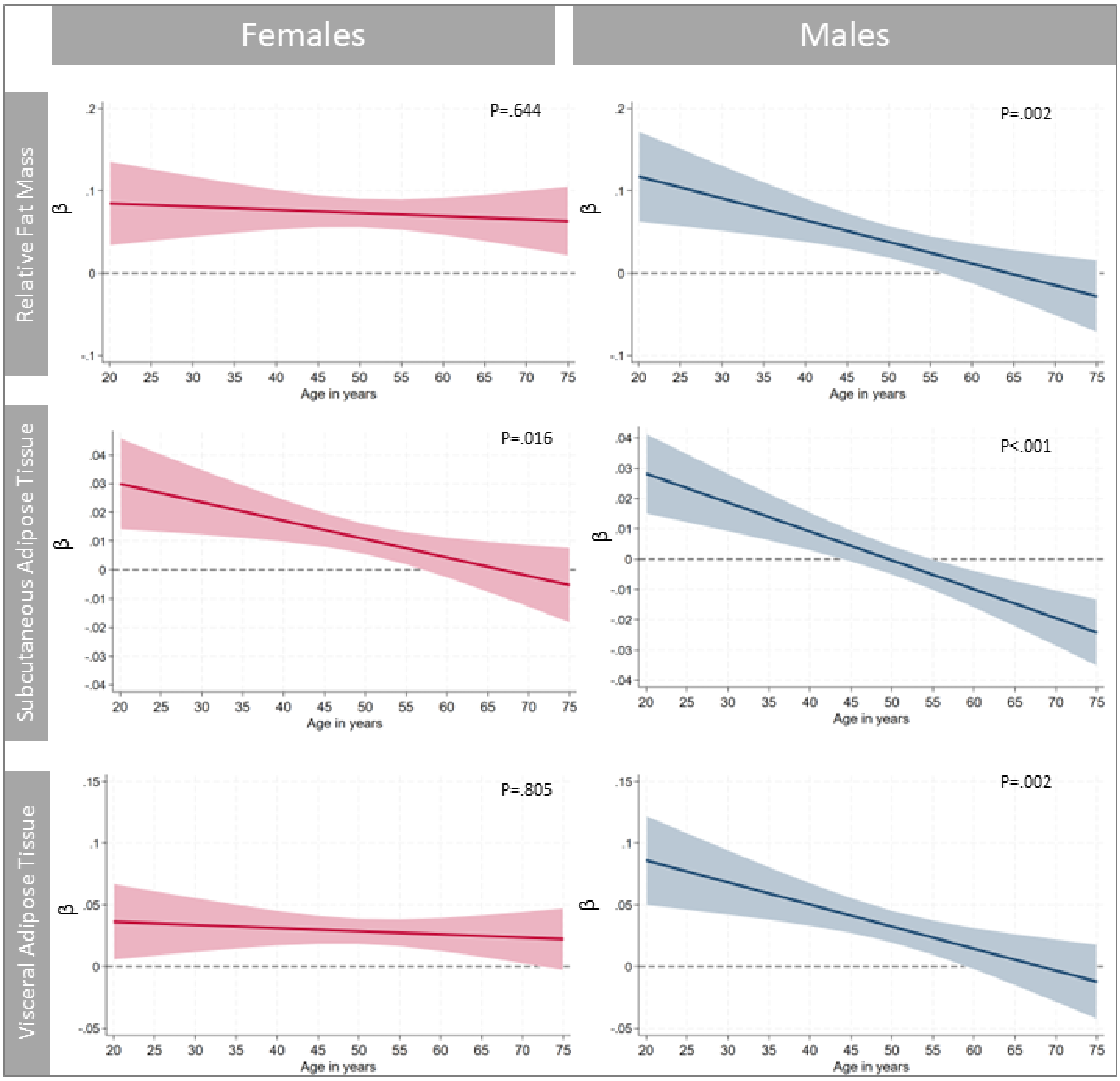

Figure S3: Age-dependent associations between CTS sum score and body fat markers for females (left) and men (right).

Note: Data are shown as β coefficients and 95% confidence intervals (shaded areas) indicating the age-dependent association between CTS sum scores and different body fat markers (i.e., relative fat mass, subcutaneous adipose tissue, and visceral adipose tissue) derived from fully adjusted sex-stratified linear regression models.

p-values denote significance levels for the CTS x age interaction.

Table S1. Sex-stratified associations of total and cumulative childhood maltreatment exposure with anthropometric and body fat markers (additionally adjusted for migration background)

Outcome	CTS sum score	1 vs. no CM	2 vs. no CM	3+ vs. no CM
**Body height in cm**				
Males	-0.06 (-0.08; -0.04)*	-0.29 (-0.43; -0.17)*	-0.15 (-0.39; 0.08)	-0.53 (-0.85; -0.22)*
Females	-0.02 (-0.03; 0.00)	0.02 (-0.10; 0.14)	-0.28 (-0.48; -0.09)	-0.04 (-0.27; 0.18)
**Body weight in kg**				
Males	0.11 (0.06; 0.15)*	0.31 (0.02; 0.60)	1.06 (0.55; 1.57)*	1.52 (0.83; 2.23)*
Females	0.21 (0.17; 0.24)*	0.57 (0.28; 0.85)*	1.49 (1.03; 1.95)*	2.98 (2.44; 3.50)*
**Body mass index in kg/m²**				
Males	0.05 (0.04; 0.06)*	0.18 (0.10; 0.27)*	0.36 (0.21; 0.50)*	0.64 (0.44; 0.84)*
Females	0.08 (0.07; 0.09)*	0.19 (0.09; 0.29)*	0.63 (0.46; 0.79)*	1.11 (0.93; 1.30)*
**Waist circumference in cm**				
Males	0.14 (0.10; 0.17)*	0.39 (0.16; 0.62)*	1.12 (0.71; 1.52)*	1.62 (1.07; 2.17)*
Females	0.22 (0.18; 0.25)*	0.54 (0.31; 0.78)*	1.67 (1.28; 2.06)*	2.75 (2.30; 3.19)*
**Waist to height ratio in %**				
Males	0.09 (0.07; 0.11)*	0.29 (0.16; 0.42)*	0.66 (0.43; 0.89)*	1.06 (0.75; 1.37)*
Females	0.13 (0.12; 0.15)*	0.31 (0.17; 0.46)*	1.09 (0.85; 1.33)*	1.66 (1.39; 1.93)*
**Relative fat mass in %**				
Males	0.03 (0.01; 0.05)*	0.13 (0.00; 0.26)	0.27 (0.04; 0.50)	0.62 (0.31; 0.93)*
Females	0.07 (0.05; 0.09)*	0.11 (-0.02; 0.24)	0.56 (0.34; 0.78)*	1.14 (0.89; 1.39)*
**Subcutaneous adipose tissue in mm**				
Males	-0.02 (-0.06; 0.03)	0.07 (-0.24; 0.38)	-0.38 (-0.95; 0.18)	0.11 (-0.70; 0.91)
Females	0.10 (0.04; 0.15)*	0.31 (-0.09; 0.70)	0.79 (0.12; 1.46)	1.29 (0.52; 2.07)*
**Visceral adipose tissue in mm**				
Males	0.31 (0.17; 0.44)*	1.26 (0.40; 2.11)	1.27 (-0.29; 2.83)	3.12 (0.92; 5.31)
Females	0.27 (0.16; 0.38)*	0.89 (0.12; 1.66)	2.61 (1.30; 3.91)*	2.54 (1.03; 4.05)*

Note: Data are reported as β coefficients and 95% confidence intervals derived from sex-stratified linear regression models adjusted for age, years of formal education, migration background, and examination center. **Abbreviations:** CM = Childhood Maltreatment; CTS = Childhood Trauma Screener.
*significant after Bonferroni correction ($p<0.003$).

Table S2. Sex-stratified associations of subtype-specific childhood maltreatment with anthropometric and body fat markers

Outcome	Emotional neglect	Physical neglect	Emotional abuse	Physical abuse	Sexual abuse
**Body height in cm**					
Males	-0.50 (-0.70; -0.31)*	-0.65 (-0.82; -0.49)*	-0.23 (-0.43; -0.03)	-0.12 (-0.30; 0.05)	-0.31 (-0.62; -0.01)
Females	-0.12 (-0.28; 0.05)	-0.52 (-0.68; -0.37)*	0.01 (-0.14; 0.16)	-0.09 (-0.26; 0.08)	0.04 (-0.11; 0.20)
**Body weight in kg**					
Males	-0.16 (-0.60; 0.27)	-0.28 (-0.64; 0.08)	1.53 (1.09; 1.97)*	1.77 (1.38; 2.15)*	0.36 (-0.32; 1.03)
Females	1.18 (0.79; 1.57)*	-0.33 (-0.69; 0.03)	2.06 (1.70; 2.42)*	2.53 (2.13; 2.93)*	1.73 (1.36; 2.09)*
**Body mass index in kg/m^2**					
Males	0.09 (-0.03; 0.22)	0.11 (0.01; 0.21)	0.54 (0.41; 0.67)*	0.58 (0.47; 0.69)*	0.21 (0.02; 0.40)
Females	0.48 (0.34; 0.61)*	0.05 (-0.08; 0.18)	0.74 (0.62; 0.87)*	0.94 (0.80; 1.08)*	0.61 (0.48; 0.74)*
**Waist circumference in cm**					
Males	0.27 (-0.06;0.61)	0.04 (-0.24; 0.33)	1.53 (1.18; 1.87)*	1.46 (1.16; 1.76)*	0.48 (-0.05; 1.01)
Females	1.30 (0.97; 1.62)*	0.14 (-0.16; 0.45)	1.96 (1.66; 2.26)*	2.28 (1.94; 2.62)*	1.51 (1.20; 1.81)*
**Waist to height ratio in %**					
Males	0.29 (0.10; 0.48)	0.22 (0.06; 0.38)	0.91 (0.72; 1.10)*	0.84 (0.67; 1.01)*	0.35 (0.05; 0.65)
Females	0.81 (0.61; 1.01)*	0.25 (0.07; 0.44)	1.17 (0.99; 1.36)*	1.39 (1.18; 1.60)*	0.89 (0.70; 1.08)*
**Relative fat mass in %**					
Males	0.03 (-0.16; 0.22)	0.08 (-0.07; 0.24)	0.56 (0.37; 0.75)*	0.49 (0.32; 0.66)*	0.21 (-0.08; 0.51)
Females	0.43 (0.25; 0.62)*	-0.02 (-0.20; 0.15)	0.79 (0.61; 0.96)*	1.00 (0.81; 1.19)*	0.58 (0.41; 0.75)*
**Subcutaneous adipose tissue in mm**					
Males	-0.11 (-0.59; 0.37)	-0.13 (-0.52; 0.26)	0.11 (-0.38; 0.59)	-0.03 (0.45; 0.40)	0.16 (-0.59; 0.91)
Females	0.52 (-0.04; 1.09)	-0.19 (-0.71; 0.33)	1.00 (0.48; 1.52)*	1.28 (0.70; 1.86)*	0.62 (0.10; 1.15)
**Visceral adipose tissue in mm**					
Males	0.91 (-0.41; 2.23)	0.92 (-0.17; 2.00)	2.35 (1.03; 3.66)*	2.37 (1.20; 3.53)*	0.25 (-1.81; 2.31)
Females	1.99 (0.88; 3.09)*	0.41 (-0.60; 1.42)	1.98 (0.97; 2.99)*	2.10 (0.97; 3.23)*	1.96 (0.95; 2.97)*

Note: Data are reported as β coefficients and 95% confidence intervals derived from sex-stratified linear regression models adjusted for age, years of formal education, and examination center.

*significant after Bonferroni correction ($p<0.003$)

Table S3. Sex-stratified associations of subtype-specific childhood maltreatment with anthropometric and body fat markers (additionally adjusted for migration background)

Outcome	Emotional neglect	Physical neglect	Emotional abuse	Physical abuse	Sexual abuse
**Body height in cm**					
Males	-0. 34 (-0.54; -0.15)*	-0.52 (-0.69; -0.36)*	-0.14 (-0.33; 0.06)	-0.01 (-0.19; 0.16)	-0.21 (-0.51; 0.10)
Females	-0.03 (-0.19; 0.13)	-0.45 (-0.61; -0.30)*	0.08 (-0.07; 0.23)	0.02 (-0.15; 0.19)	0.06 (-0.09; 0.21)
**Body weight in kg**					
Males	-0.09 (-0.52; 0.35)	-0.21 (-0.57; 0.15)	1.58 (1.13; 2.02)*	1.82 (1.43; 2.21)*	0.39 (-0.29; 1.07)
Females	1.23 (0.84; 1.62)*	-0.28 (-0.65; 0.08)	2.11 (1.75; 2.47)*	2.61 (2.21; 3.01)*	1.74 (1.37; 2.10)*
**Body mass index in kg/m²**					
Males	0.07 (-0.05; 0.19)	0.09 (-0.01; 0.20)	0.53 (0.40; 0.65)*	0.56 (0.45; 0.67)*	0.18 (-0.01; 0.38)
Females	0.47 (0.33; 0.61)*	0.05 (-0.08; 0.17)	0.74 (0.61; 0.87)*	0.94 (0.80; 1.08)*	0.61 (0.48; 0.74)*
**Waist circumference in cm**					
Males	0.28 (-0.06;0.62)	0.05 (-0.22; 0.34)	1.53 (1.18; 1.88)*	1.47 (1.16; 1.77)*	0.48 (-0.06; 1.00)
Females	1.31 (0.98; 1.63)*	0.15 (-0.15; 0.46)	1.97 (1.67; 2.28)*	2.30 (1.96; 2.64)*	1.51 (1.20; 1.82)*
**Waist to height ratio in %**					
Males	0.25 (0.06; 0.44)	0.18 (0.03; 0.34)	0.88 (0.69; 1.08)*	0.81 (0.64; 0.98)*	0.31 (0.02; 0.61)
Females	0.79 (0.59; 0.99)*	0.24 (0.05; 0.44)	1.16 (0.97; 1.34)*	1.37 (1.16; 1.58)*	0.89 (0.70; 1.07)*
**Relative fat mass in %**					
Males	0.00 (-0.19; 0.19)	0.06 (-0.10; 0.22)	0.54 (0.35; 0.74)*	0.46 (0.29; 0.63)*	0.19 (-0.11; 0.48)
Females	0.41 (0.23; 0.60)*	-0.03 (-0.21; 0.14)	0.77 (0.60; 0.94)*	0.98 (0.79; 1.17)*	0.58 (0.41; 0.75)*
**Subcutaneous adipose tissue in mm**					
Males	-0.13 (-0.61; 0.35)	-0.15 (-0.54; 0.25)	0.09 (-0.39; 0.57)	-0.04 (0.47; 0.38)	0.14 (-0.61; 0.89)
Females	0.50 (-0.06; 1.07)	-0.20 (-0.72; 0.32)	0.98 (0.47; 1.51)*	1.26 (0.68; 1.84)*	0.62 (0.10; 1.14)
**Visceral adipose tissue in mm**					
Males	0.87 (-0.45; 2.19)	0.87 (-0.21; 1.96)	2.31 (1.00; 3.63)*	2.33 (1.17; 3.50)*	0.22 (-1.84; 2.27)
Females	1.92 (0.82; 3.03)*	0.38 (-0.63; 1.39)	1.95 (0.94; 2.96)*	2.05 (0.92; 3.18)*	1.94 (0.93; 2.96)*

Note: Data are reported as β coefficients and 95% confidence intervals derived from sex-stratified linear regression models adjusted for age, years of formal education, migration background, and examination center.

*significant after Bonferroni correction ($p<0.003$)
